# FM-DLM: A new method for image classification based on the fusion of multi-level deep learning models

**DOI:** 10.1371/journal.pone.0338137

**Published:** 2026-01-27

**Authors:** Guanghao Jin, Hengguang Li, Hui Du, Qingzeng Song

**Affiliations:** 1 School of Artificial Intelligence, Beijing Polytechnic, Beijing, China; 2 Department of Mathematics, Wayne State University, Detroit, Michigan, United States of America; 3 School of Computer Science and Technology, Tiangong University, Tianjin, China; Universidade Federal de Uberlandia, BRAZIL

## Abstract

Currently, deep learning models are widely used in many classification applications, but their utilization is limited by some factors. The large models can ensure classification of wide range, but they cannot be deployed to some small devices. The small models can be deployed to the small devices, but the number of labels is limited. To solve these problems, this paper proposes a classification method based on the Fusion of Multi-level Deep Learning Models (FM-DLM). We apply the Baidu-AI platform as a Level 0 model for classification of wide range samples. Then, we use the difference between Level 1 models to perform dataset prediction. Then, we can use the Level 2 models that were trained on the predicted dataset, which is to perform label classification. Finally, we use label distribution to achieve higher accuracy. The experimental results show that our method can achieve higher accuracy than the existing methods while ensuring a wide range of classification.

## Introduction

With the development of deep learning technology, various models are constantly being designed [1,2]. The current development trend is design large models, which requires more computing resources and leads to high training cost [3,4]. Therefore, some small devices may not be able to implement these large models.

With the emergence of commercial large model platforms, we can perform the classification task by calling APIs (Application Programming Interfaces) [5,6]. Although these platforms can provide the classification service through the network, the training of new samples is not convenient, which causes low classification accuracy on these samples.

The fusion of deep learning models is another solution. To increase accuracy, some methods fuse the output of multiple models [7,8]. On the other hand, the accuracy depends on the output of each model. Therefore, the selection of high-precision models is the decisive factor. In addition, if these models are trained on different datasets, we should first predict the dataset.

To address these challenges, we try to efficiently utilize a large model platform and the fusion of deep learning models to achieve a wide range of classification and high accuracy. The contributions of this paper can be summarized as follows. The first one is that we efficiently match the result of the large model to the label of the datasets, which ensures a wide range of classification. The second is that our method optimizes the fusion methods, which achieves dataset prediction while ensuring high accuracy. The third one is that our method can be deployed to different types of small devices, which solves the problem of device dependency.

The structure of our paper is as follows. The first section is the introduction and the second one is related work. The third section introduces our method and the fourth one introduces the experiment. The fifth section summarizes this paper and discusses future work.

## Related work

In this paper, we choose some existing deep learning models, large model platform and fusion methods as the baseline. The first type of deep models is based on SNN (Spiking Neural Networks) mechanism. The second type of deep models include others that have different kinds of structures. We introduce a large model platform that will be utilized at our Level 0. Then, we introduce some fusion methods that will be utilized at the other levels.

The first type of deep learning models is based on SNN. The ANN-SNN model applies the quantization clip-floor-shift activation function to replace the ReLU (‌Rectified Linear Unit), which can better approximate the activation function [9]. Hybrid training SNN (Spiking Neural Networks) utilizes a computationally efficient training technique [10]. The Low-Latency SNN model proposes a low-latency deep spiking network trained with gradient descent, which optimizes the membrane leak and the firing threshold [11]. The direct training SNN model proposes a neuron normalization technique to adjust neural selectivity and develops a direct learning algorithm for deep SNNs [12]. The TSSL-BP (Temporal Spike Sequence Learning Back Propagation) model uses a novel temporal spike sequence learning back propagation method for training deep SNNs [13]. The TDBN (Threshold Dependent Batch Normalization) model enables direct training of a very deep SNN and the efficient implementation of its inference on hardware [14]. These models utilize different optimization techniques based on the structure of the SNN. Thus, the performance is limited by the structure of the SNN.

We introduce some different deep learning models to expand the range of model selection in our method case. The TET (Temporal Efficient Training) model introduces a temporal efficient training approach to compensate for the loss of momentum in gradient descent [15]. The MPD (Membrane Potential Distribution) model attempts to rectify the membrane potential distribution by designing a novel distribution loss, which can explicitly penalize the undesired shifts without introducing any additional operations in the inference phase [16]. The WRN (Wide Residual Networks) model proposes a ground radar target classification algorithm and an attention mechanism [17]. The DeiT (Data-efficient image Transformers) model produces a competitive convolution-free transformer by training only on ImageNet [18]. The Swin (Shifted Window) model presents a new vision transformer that capably serves as a general-purpose backbone for computer vision [19]. These models utilize different structures and parameter tuning techniques, which achieve high accuracy on various datasets. On the other hand, there are no models that can achieve the highest accuracy on all datasets. Thus, we try to efficiently utilize these models to achieve high accuracy on multiple datasets.

To enable a wide range of classification, we select the Baidu-AI platform as the large model platform [20]. This platform can identify more than 100,000 kinds of objects and scenes, and provide corresponding API services to fully meet the application needs of various developers and enterprise users. The API request is used for general object and scene recognition; that is, for an input picture (which can be decoded normally and has an appropriate aspect ratio), multiple object and scene labels in the picture are output.

To efficiently use multiple models to achieve high accuracy, we choose some of the latest fusion methods. These methods can fuse the outputs of models on multiple datasets. Voting methods combine the top-performing models to achieve the high accuracy [21]. Weighted voting methods try to fuse various deep neural network models to achieve high accuracy [22].

We summarize these methods in Table 1. Basically, these are summarized as three types,

**Table 1:** Literature table of related work.

Paper ID	Type	Advantage	Limitation
[9–19]	Single models	Easy to train	Limited range of classification
[20]	Large model platforms	Wide range classification	High cost of training.
[21,22]	Fusion methods	Accurate label classification	Affected by the low accurate models

single models, large model platforms and fusion methods. We introduce the advantages and limitations of these methods in this table.

## Our method

In order to help understand the methods in this paper, we first provide some definitions.

### Preliminaries

Firstly, we define *S*_*n*_ as a sample and *G*(*S*_*n*_) as the ground truth of *S*_*n*_. We name a dataset to be *D*_*j*_. Then, we define a deep learning model *M*_*i*_ that is trained on *D*_*j*_ as *M*_*i,j*_. We define the output of model *M*_*i,j*_ on *S*_*n*_ as *F* (*M*_*i,j*_, *S*_*n*_). Then, we can define the accuracy of *M*_*i,j*_ on {*S*_*n*_} as $P_{S_{n}\in \{S_{n}\}}\left({F(M}_{i,j},S_{n})=G\left(S_{n}\right)\right)$. On some samples, if the model with the highest classification accuracy is *M*_*i,j*_, we define these samples to belong to *D*_*j*_. We define *L*_*k, j*_ as a label of *D*_*j*_. We define $N\left({\{L}_{k,j}\}\right)$ is the number of labels in {*L*_*k, j*_}. Then, we construct the levels by the following equation.


$$P_{S_{n}\in D_{j}}\left({F(M}_{\vec{i},\vec{j}},S_{n})=G\left(S_{n}\right)\right)<P_{S_{n}\in D_{j}}\left({F(M}_{i,j},S_{n})=G\left(S_{n}\right)\right)$$

$$N\left(\{L_{\vec{k},\vec{j}}\}\right)>N\left(\{L_{k,j}\}\right).$$ (1)

where $N\left(\{L_{\vec{k},\vec{j}}\}\right)$ represents the number of labels in Level $\vec{j}$ and $N\left(\{L_{k,j}\}\right)$ is the number of layers at Level *j*. Equation 1 represents the relationship between models in different levels. Lower-level models have more labels than higher-level models. On the contrary, due to the targeted training of corresponding datasets, we assume that the higher-level model achieves higher accuracy.

We can define the classification task in this paper as follows. When there are multiple datasets {*D*_*j*_}, a classification process should first predict which *D*_*y*_ includes *S*_*n*_. Then, on the predicted dataset *D*_*y*_, it should classify the label *L*_*x,y*_ of *S*_*n*_.

### Our framework

**Fig 1:**
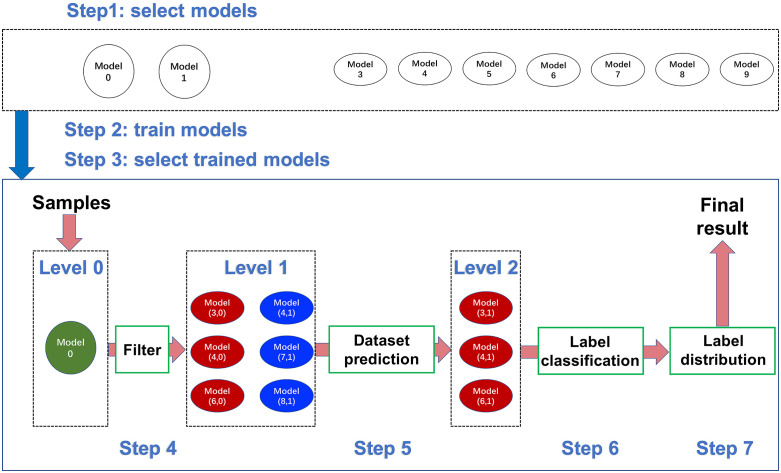
The framework our method.

In this subsection, we illustrate our method (named FM-DLM) as shown in Fig 1. Step 1 is the selection of models, including the selection of a large model platform, based on whether they are shared and can be deployed to our devices. Step 2 is model training, which follows the training process outlined in the relevant papers. Step 3 is model selection, mainly selecting some trained models with higher accuracy on the corresponding dataset.

After these preparations, we can utilize our method to classify the samples. Step 4 uses the large model platform (named as the Level 0 model) to classify the labels of samples. Then, our method filters the label from Level 0 to that of Level 1, which is used to temporally predict the dataset. Step 5 aims to predict the dataset more precisely according to the differences among Level 1 models. Step 6 selects the trained models (named Level 2 models) of the predicted dataset and fuse their outputs to classify the label. Step 7 uses the label distribution to optimize the results, which further increases classification accuracy.

In practical applications, each device can choose the appropriate type and number of models based on storage and hard disk capacity. When we use the model platform at Level 0, we only need to ensure the internet connection. When we select the Level 1 models, we can choose the appropriate number of models according to memory capacity. Then, we can also run the models one by one in memory, which is suitable for small memory sizes. We summarize the levels as shown in Table 2.

**Table 2:** The introduction of the levels.

Level		Role	Advantage	Limitation
Level 0	A large model platform like Baidu-AI	Decide whether a sample belongs to the collected datasets or not	Wide range classification	Low accuracy
Level 1	Multiple models	Dataset prediction	Easy to train the high accurate models	More datasets reduce the accuracy
Level 2	Multiple models on corresponding dataset	Label classification	Accurate label classification	Consumes more execution time

### Step 1: Select models

Firstly, we collect some models based on the following conditions. The first condition is that these models should be open-source and shareable. The second condition is that the memory consumption of these models is smaller than that of our GPU. The third condition is that we can deploy these models without any bug.

We train these models following the training steps that are introduced in the related papers. Some models may have low performance on datasets that are not introduced in these papers. In other words, the tuning of these models is highly related to the corresponding datasets. Thus, the selection of trained models in Step 3 is important.

### Step 2: Train models

If we use a commercial large model platform as Level 0 model, we don’t need model training at this level. These large model platforms can classify a wide range of labels.

When training the models at Level 1, we first prepare some existing models {*M*_*i*_} and public datasets {*D*_*j*_}. We select each dataset *D*_*j*_ and divide it into a training set ${\overset{―}{D}}_{j}$, a validation one ${\overset{ˇ}{D}}_{j}$ and a testing one ${\tilde{D}}_{j}$. Then, each model *M*_*i*_ is trained on ${\overset{―}{D}}_{j}$ to obtain a trained model *M*_*i,j*_. When we train *M*_*i*_ on another *D*_*jj*_, we can get a trained model *M*_*i,jj*_. Each model is trained on one dataset in our method.

### Step 3: Select trained models

The rule for selecting trained models is based on their accuracy on the validation sets. We choose some models {*M*_*x,j*_} with higher accuracy among the trained models on each dataset as follows.


$$\{{M_{x,j}\}={Top}_{i}P}_{S_{n}\in {\overset{ˇ}{D}}_{j}}\left({F(M}_{i,j},S_{n})=G\left(S_{n}\right)\right),\;$$ (2)

where $P_{S_{n}\in {\overset{ˇ}{D}}_{j}}\left({F(M}_{i,j},S_{n})=G\left(S_{n}\right)\right)$ is defined by Equation 1 and *Top*_*i*_ means we select the top *i* models that achieved higher accuracy than the others on the validation set. The number *i* is also determined by the validation set.

### Step 4: Filter

We can classify the label of a sample by Level 0 model. If the classified label belongs to the collected datasets {*D*_*j*_}, we can continue to the next process. Otherwise, the classified label is the final result. The large model platform has a relatively wide distribution of labels, but the label format follows its own rules. Therefore, there are some differences between the labels provided by the large model platform and those of {*D*_*j*_}. Thus, we should map the labels of Level 0 to those of Level 1. We can define the mapping as follows.


$$\begin{array}{c} {{P\left(L_{\vec{k}},L_{k,j}\right)=P}_{S_{n}\in D_{j}}\left({F(M}_{\text{0}},S_{n})=L_{\vec{k}}\right)/P\;}_{S_{n}\in D_{j}}\left(G\left(S_{n}\right)=L_{k,j}\right) \end{array}$$ (3)

where $L_{\vec{k}}$ is the label of Level 0 and *L*_*k,j*_ is the label of Level 1. $P_{S_{n}\in D_{j}}\left({F(M}_{\text{0}},S_{n})=L_{\vec{k}}\right)$ is defined by Equation 1, and $P_{S_{n}\in D_{j}}\left(G\left(S_{n}\right)=L_{k,j}\right)$ is the probability when the ground truth *G*(*S*_*n*_) is the label *L*_*k,j*_.

### Step 5: Dataset prediction

When we classify *S*_*n*_ that belongs to a certain dataset by Step 4, we should predict which dataset contains this sample. We use the difference among Level 1 models to classify the dataset that may include this sample.

We define the probability of a label *L*_*k, j*_ on a sample *S*_*n*_ by the output of *M*_*i,j*_ as *P* (*M*_*i,j*_, *S*_*n*_, *L*_*k,j*_). On the validation set, we can get the weights {*W*_*i,j*_} as shown in the following equation.


$$W_{i,j}=\frac{\text{1}/(\text{1}-P\left(L_{k,j}=G\left(S_{n}\right),\;S_{n}\in {\overset{ˇ}{D}}_{j}\right)}{\sum_{{\overset{ˇ}{D}}_{j}}\text{1}/(\text{1}-P\left(L_{k,j}=G\left(S_{n}\right),\;S_{n}\in {\overset{ˇ}{D}}_{j}\right)},$$ (4)

where here $P\left(L_{k,j}=G\left(S_{n}\right),\;S_{n}\in {\overset{ˇ}{D}}_{j}\right)$ is defined as the accuracy of trained model *M*_*i,j*_ on the validation set *D*_*j*_. We define the difference between *M*_*i,j*_ with *M*_*ii,j*_ on a sample *S*_*n*_ as follows.


$$\left\|M_{i,j}-M_{ii,j}\right\|=\sum {\mathrm{\backslash nolimits}}_{L_{k,j}}\left|{P(M}_{i,j},S_{n},L_{k,j})\times W_{i,j}-{P(M}_{ii,j},S_{n},L_{k,j})\times W_{ii,j}\right|,$$ (5)

where *P* (*M*_*i,j*_, *S*_*n*_, *L*_*k,j*_), *W*_*i,j*_ and *W*_*ii,j*_ are defined by Equation 4. *W*_*i,j*_ is the weight that is related to model *M*_*i,j*_ and *W*_*ii,j*_ is the weight that is related to model *M*_*ii,j*_. We can select *M*_*ii,j*_ that achieve the highest accuracy on ${\overset{ˇ}{D}}_{j}$. Then, we can predict the dataset that may include *S*_*n*_ by the following equation.


$$D_{y}={argmin}_{j}\sum {\mathrm{\backslash nolimits}}_{i}\left\|M_{i,j}-M_{ii,j}\right\|,$$ (6)

where we define *D*_*y*_ as the predicted dataset that may contain the sample *S*_*n*_.

### Step 6: Label classification

After the *D*_*y*_ is obtained through Equation 6, we can classify the labels by the following equation:


$$L_{x,y}={argmin}_{k}\sum {\mathrm{\backslash nolimits}}_{i}W_{i,y}\times {P(M}_{i,y},S_{n},L_{k,y}),$$ (7)

where *W*_*i,y*_ and *P* (*M*_*i,y*_*, S*_*n*_*, L*_*k,y*_) are introduced in Equation 4. *P* (*M*_*i,y*_*, S*_*n*_*, L*_*k,y*_) is the output of the trained models of *D*_*y*_.

### Step 7: Label distribution

Generally, a validation set is used to simulate the corresponding testing set. Therefore, we can assume that the label distribution of the validation set is the same as that of the testing set. We compute the distribution of label *L*_*k,j*_ on dataset ${\overset{ˇ}{D}}_{j}$ using the following equation.


$$P\left(L_{k,j},{\overset{ˇ}{D}}_{j}\right)=\frac{\sum_{S_{n}\in {\overset{ˇ}{D}}_{j}\;G\left(S_{n}\right)=L_{k,j}}\text{1}}{\sum_{S_{n}\in {\overset{ˇ}{D}}_{j}}\text{1}}.$$ (8)

where $\sum_{S_{n}\in {\overset{ˇ}{D}}_{j},\;G\left(S_{n}\right)=L_{k,j}}\text{1}$ presents the number of samples (that belong to ${\overset{ˇ}{D}}_{j}$), for which the ground truth is *L*_*k,j*_. $\sum_{S_{n}\in {\overset{ˇ}{D}}_{j}}\text{1}$ is the number of all samples that belong to ${\overset{ˇ}{D}}_{j}$. After the labels are classified by Equation 7, the scores of some results may be low. We can set thresholds based on the validation set to select some of these results. For these results, we further increase the accuracy using the following equation:


$${\tilde{L}}_{x,y}={argmin}_{k}\sum_{i}W_{i,y}\times {P(M}_{i,y},S_{n},L_{k,y})+\omega \times P\left(L_{k,y},{\overset{ˇ}{D}}_{\text{y}}\right)$$ (9)

where *P* (*M*_*i,y*_*, S*_*n*_*, L*_*k,y*_) is defined by Equation 4 and $P\left(L_{k,y},{\overset{ˇ}{D}}_{\text{y}}\right)$ is defined by Equation 8. We also compute the hyper-parameter ω on the validation set.

### Meticulous pseudo code related to our method

We introduce the pseudo code related to our method in Table 3.

**Table 3:** Pseudo code of FM-DLM.

**//Step 1: select models** **Select** the models {*M*_*i*_} and the datasets {*D*_*j*_}. **//Step 2: train models** **Separate** the dataset *D*_*j*_ into a training set ${\overset{―}{D}}_{j}$, a validation set ${\overset{ˇ}{D}}_{j}$, and a testing set ${\tilde{D}}_{j}$. **For** ${\overset{―}{D}}_{j}$ in ${\;\{\overset{―}{D}}_{j}\}$ **For** *M*_*i*_ in {*M*_*i*_} **Train** *M*_*i*_ on ${\overset{―}{D}}_{j}$ to get a trained model *M*_*i,j*_. **//Step 3: select trained models** **For** *M*_*i,j*_ in {*M*_*i,j*_} **Get** ${{\{M}_{x,j}\}={Top}_{i}P}_{S_{n}\in {\overset{ˇ}{D}}_{j}}\left({F(M}_{i,j},S_{n})=G\left(S_{n}\right)\right)$ by Equation 2. **//Step 4: filter** **Between** Level 0 and Level 1 **Compute** $P\left(L_{\vec{k}},L_{k,j}\right)$ by Equation 3.
**//Step 5: dataset prediction** **For** *j* from 0 to the number of datasets **For** *i* from 0 to the number of models **Compute** *W*_*i,j*_ by Equation 4. **For** $S_{n}\in \{S_{n}\}$ Use *M*_0_ of Level 0 to get the label $L_{\vec{k}}$ of this sample. **If** $P\left(L_{\vec{k}},L_{k,j}\right)>\text{0}$ and $L_{k,j}\in \{D_{j}\}$ **Compute** the *D*_*y*_ by Equations 5 and 6. **//Step 6: label classification** **Compute** *L*_*x,y*_ by Equation 7. **//Step 7: label distribution** **Compute** ${\tilde{L}}_{x,y}$ by Equations 8 and 9. **Output** ${\tilde{L}}_{x,y}$ as the final result. **Else** **Output** $L_{\vec{k}}$ as the final result.

## Experiment

### Experimental setup

We selected three public datasets: the CIFAR-10 dataset [23], the CIFAR-100 dataset [24], and the Mini-ImageNet dataset [25]. Generally, for each dataset, we use 70% samples for training and 10% of validation, and 20% for testing.

We select some shared models, which are ANN-SNN [9], Hybrid training SNN [10], Low-latency SNN [11], Direct training SNN [12], TSSL-BP [13], TDBN [14], TET [15], MPD [16], WRN [17], DeiT [18], Swin (Shifted Window) [19]. The selection depends on the possibility of implementation on our device. We select Baidu-AI large model platform as the Level 0 model. Table 4 shows the details of classification by Baidu-AI.

**Table 4:** The introduction of Baidu-AI.

Return parameters	Values	Types	Explanation
Result_num	5	unit32	The number of returned results and the number of elements in the result array
Result	Text	array(object)	Label result array
Score	Text	float	Confidence (0–1)
Keyword	Text	string	Name of object or scene in the picture

For better comprehension, we use Table 5 to explain the evaluation metrics.

**Table 5:** The experimental process of our method (FM-DLM).

**Separate** a dataset *D*_*j*_ into a training set ${\overset{―}{D}}_{j}$, a validation set ${\overset{ˇ}{D}}_{j}$, and a testing set ${\tilde{D}}_{j}$. **// Accuracy of label classification** $P_{S_{n}\in {\tilde{D}}_{j}}\left({F(M}_{i,j},S_{n})=G\left(S_{n}\right)\right)=\frac{\sum_{S_{n}\in {\tilde{D}}_{j},{F(M}_{i,j},S_{n})=G\left(S_{n}\right)}\text{1}}{\sum_{S_{n}\in {\tilde{D}}_{j}}\text{1}}$, (10) where $S_{n}\in {\tilde{D}}_{j}$ means a sample *S*_*n*_ in the dataset ${\tilde{D}}_{j}$, ${F(M}_{i,j},S_{n})$ is the classified label by a model *M*_*i,j*_ on the *S*_*n*_. *G* (*S*_*n*_) is the ground truth of *S*_*n*_. **// Accuracy of dataset prediction** $P_{S_{n}\in {\tilde{D}}_{j}}\left(D_{y}={\tilde{D}}_{j}\right)=\frac{\sum_{S_{n}\in {\tilde{D}}_{j},,D_{y}={\tilde{D}}_{j}}\text{1}}{\sum_{S_{n}\in {\tilde{D}}_{j}}\text{1}}$, (11) where $S_{n}\in {\tilde{D}}_{j}$ means a sample *S*_*n*_ in the dataset ${\tilde{D}}_{j}$, *D*_*y*_ is the dataset that is predicted by the methods.
**// Accuracy of label classification with dataset prediction** $P_{S_{n}\in {\tilde{D}}_{j}}\left({F(M}_{i,j},S_{n})=G\left(S_{n}\right),D_{y}={\tilde{D}}_{j}\right)=\frac{\sum_{S_{n}\in {\tilde{D}}_{j},{F(M}_{i,j},S_{n})=G\left(S_{n}\right),D_{y}={\tilde{D}}_{j}}\text{1}}{\sum_{S_{n}\in {\tilde{D}}_{j}}\text{1}}$, (12) where $S_{n}\in {\tilde{D}}_{j}$ means a sample *S*_*n*_ in the dataset ${\tilde{D}}_{j}$, *F*(*M*_*i,j*_, *S*_*n*_) is the classified label by a model *M*_*i,j*_ on the *S*_*n*_. *G* (*S*_*n*_) is the ground truth of *S*_*n*_. *D*_*y*_ is the dataset that is predicted by the methods.

### The evaluation of trained models (Step 3)

Table 6 shows the classification accuracy of different models on three public datasets. Due to the different optimization details, the accuracy of these models is different on various data sets. We build our framework based on the selection of these models at Step 3.

**Table 6:** The evaluation of trained models (accuracy of label classification, percentage).

Models	Dataset		
	CIFAR-10	CIFAR-100	Mini-ImageNet
ANN-SNN [9]	90.42	75.69	85.15
Hybrid training SNN [10]	92.19	67.88	83.14
Low-latency SNN [11]	92.49	64.09	82.13
Direct training SNN [12]	90.51	70.01	84.43
TSSL-BP [13]	91.31	71.03	85.13
TDBN [14]	93.14	72.13	86.71
TET [15]	94.48	74.71	87.00
MPD [16]	95.45	74.19	90.11
WRN [17]	96.55	80.10	94.10
DeiT [18]	99.08	91.33	95.20
Swin [19]	92.36	85.13	98.09

### The evaluation of dataset prediction (Step 5)

Table 7 shows the accuracy of the dataset prediction at Step 5. Compared with the existing methods, our method is 3.5% higher on CIFAR-10, 2.47% higher on CIFAR-100, and 3.96% higher on Mini-ImageNet than those of the existing methods.

**Table 7:** CIFAR-10, CIFAR-10 and Mini-ImageNet (accuracy of dataset prediction, percentage).

Method	CIFAR-10	CIFAR-100	Mini-ImageNet
Baidu-AI [21]	69.66	45.12	78.89
Voting [22]	81.67	68.59	82.75
Weighted voting [23]	86.18	71.85	87.82
Our method	89.68	74.32	91.78

### The evaluation of label classification with dataset prediction (Step 6)

Table 8 shows the accuracy of the label classification with of Step 6. Compared with the existing methods, our method is 4.01% higher on CIFAR-10, 3.01% higher on CIFAR-100, and 5.03% higher on Mini-ImageNet than those of the existing methods.

**Table 8:** Accuracy of label classification with dataset prediction, percentage.

Method	CIFAR-10	CIFAR-100	Mini-ImageNet
Baidu-AI	60.51	36.72	72.90
Voting	74.60	58.49	76.96
Weighted voting	82.20	64.63	84.80
Our method	86.21	67.64	89.83

### The evaluation of label distribution (Step 7)

In the label distribution step, we assigned a random distribution to the samples of labels. Table 9 shows the accuracy of the label distribution. Compared with our method (Step 6), our method with label distribution (Step 7) is 1.83% higher on CIFAR-10, 2.1% higher on CIFAR-100, and 1.5% higher on Mini-ImageNet.

**Table 9:** Accuracy of label classification with dataset prediction, percentage.

Method	CIFAR-10	CIFAR-100	Mini-ImageNet
Our method	86.21	67.64	89.83
Our method (with label distribution)	88.04	69.74	91.33

### The evaluation of ablation

Table 10 shows the ablation experimental results of the steps. From Step 1 to Step 4, the best performance achieved by the Baidu-AI. After we added dataset prediction at Step 5, we can use the corresponding model to classify the labels, which allows our method to achieve the best performance. When we fuse the outputs of models to classify labels at Step 6, our method also achieves the best performance. Furthermore, when we optimize the results using label distribution at Step 7, our method can achieve higher accuracy than that of Step 6.

**Table 10:** The evaluation of each step (accuracy of label classification with dataset prediction, percentage).

The steps	Best performance	CIFAR-10	CIFAR-100	Mini-ImageNet
From Step 1 to Step 2	Baidu-AI	60.51	36.72	72.90
From Step 1 to Step 3, (+ model selection)	Baidu-AI	60.51	36.72	72.90
From Step 1 to Step 4	Baidu-AI	60.51	36.72	72.90
From Step 1 to Step 5 (+dataset prediction)	Our method	83.07	65.43	85.05
From Step 1 to Step 6, (+label classification)	Our method	86.21	67.64	89.83
From Step 1 to Step 7, (+label distribution)	Our method	88.04	69.74	91.33

### The evaluation of the model selection

**Fig 2:**
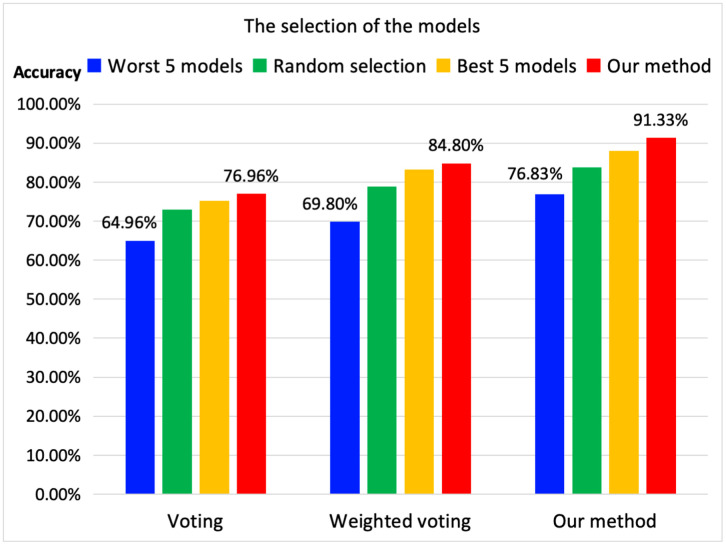
Selection of models (accuracy of label classification with dataset prediction).

Fig 2 shows how the selection of models affects the classification accuracy on Mini-ImageNet. In this figure, the blue column shows the methods with the worst (models achieve lower accuracy than those of the others) 5 trained models on each dataset. The orange column shows the methods with the best (models that achieve the highest accuracy on the corresponding dataset) trained models on the corresponding datasets. The green column shows the methods with random selection of the trained models (randomize the number of models and the selection of these models), and then we compute the average accuracy of 100 times. As this figure shows, the selection of the models play important role to the accuracy. Our method achieve the highest accuracy among all of these selections.

The voting method does not consider the importance of high-precision models, which reduces accuracy. The weighted voting method solves this problem, but it only uses the classified label, which is the final output of each model. In contrast, our method fully utilizes the probability of each label before the final output. Therefore, our method can achieve higher accuracy than other methods.

### The experiments on more datasets and models

**Fig 3:**
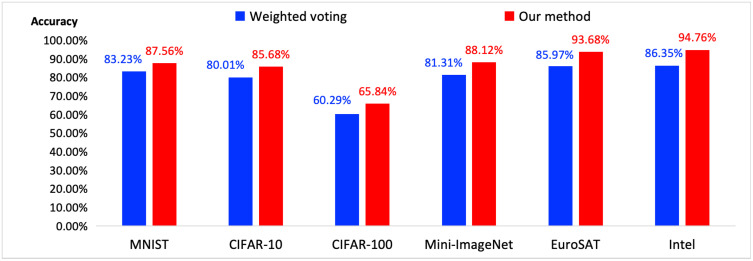
The evaluation on more datasets.

Fig 3 shows our methods on 6 datasets. With the collected 3 datasets, we further collected the EuroSAT dataset [26], Intel-image-classification dataset (named as Intel) [27] and MNIST [28,29]. Furthermore, we adopted related models for MNIST, which are F2PQNN [28] and NoRD [29]. These models achieves high accuracy on MNIST (F2PQNN is 99.09% and NoRD is 96.74%), so we adopt these models in our method. The accuracy on 6 datasets is lower than that of 3 datasets. The variety of samples is increased as there are more datasets, which reduces the accuracy of dataset prediction. Thus, label classification by Level 0 model plays important role in reducing the difficulty of dataset prediction at Level 1. Compared with the weighted voting method, our method achieved higher accuracy on each dataset.

### The analysis

**Fig 4:**
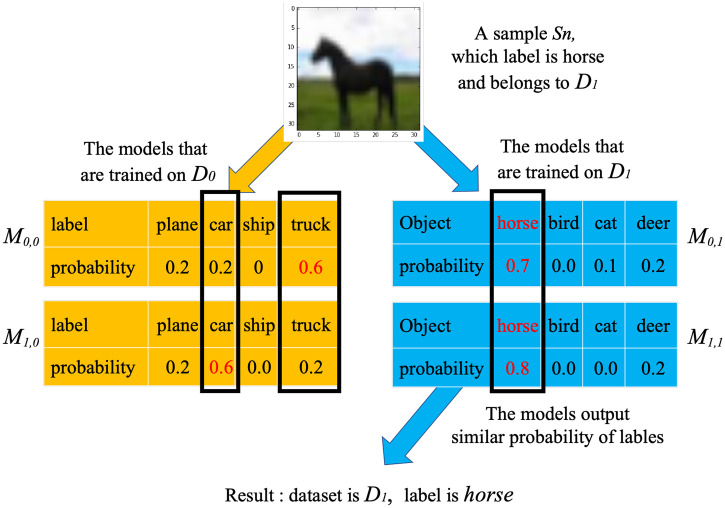
A simple illustration of our method.

Fig 4 shows a simple illustration of our method. As this figure shows, when a sample *S*_*n*_ belongs to *D*_1_, the corresponding trained models {*M*_*i,*1_} are more easily output the similar probabilities of the labels. Furthermore, the probability of ground truth will be higher than those of the others. On the other hand, as the trained models {*M*_*i,*0_} on *D*_0_ cannot effectively capture the features of this sample, it leads to different outputs of models.

### Hardware efficiency metrics

The CPU and GPU that are applied in our experiment are shown in Table 11. We select Mini-ImageNet as an example dataset. We record the maximum execution time, maximum memory consumption and FLOPs(G) of single model (we record maximum value among all models), the existing methods and our method, which are shown in Table 12. Our methods generate multiple models on each dataset, which causes the runtime to be larger than that of single model. Furthermore, the connection time with Baidu-AI consumes more time than that on either CPU or GPU.

**Table 11:** The execution time and memory consumption.

CPU	
System	Ubuntu 16.04.5 LTS
CPU	14 core Intel(R) Gold 6330 CPU @2.00GHz
memory	30G
SSD	1.8T
Python version	3.8
Pytorch	1.11.0
GPU	
GPU	RTX A5000
GPU driver	470.82.01
GPU memory	24GB
CUDA	11.3
cuDNN	7.6.3_0

**Table 12:** The execution time and memory consumption.

Method	Time				
	Mini-ImageNet (testing set, second)			Max memory consumption on GPU	FLOPs (G)
	Connection with Baidu-AI (second)	Max CPU time (second)	Max GPU time (second)		
Single model	25103	531		1813.73 MB	4.82
Voting		778	1709	1813.73 MB	4.82
Weighted voting		1062	1709	1813.73 MB	4.82
Our method		1472	1913	2087.04 MB	5.12

Compared with the execution time of single model (we record the model achieved maximum execution time), the existing fusion methods run multiple models on the GPU, which leads to longer execution time. Furthermore, these fusion methods fuse the outputs of multiple models on the CPU side, which lead to additional execution time. Our method outputs the probability of the labels on GPU side and computes the final results on CPU side, which leads to longer execution time than those of existing methods.

The existing methods and our one run the models one by one on the GPU side. Thus, the maximum memory consumption of the existing methods is the same as that of single model. Our method needs to store the probability of models, which lead to additional memory consumption.

The execution time for connecting to the Baidu-AI is the same in the cases of single models and fusion methods. Without classification by Baidu-AI, none of the methods can perform wide range classification.

### The introduction of the employed acronyms

We use Table 13 to introduce the employed acronyms in this paper.

**Table 13:** The introduction of the employed acronyms.

Number	Acronyms	Introduction
1	API	Application Programming Interface
2	ANN-SNN	Analog Neural Networks
3	SNN	Spiking Neural Networks
4	ReLU	‌Rectified Linear Unit
5	TSSL-BP	Temporal Spike Sequence Learning Back Propagation
6	TDBN	Threshold Dependent Batch Normalization
7	TET	Temporal Efficient Training
8	MPD	Membrane Potential Distribution
9	WRN	Wide Residual Networks
10	DeiT	Data-efficient image Transformers
11	Swin	Shifted Window

## Conclusions

This paper proposes a new way to efficiently utilize different levels of deep learning models to achieve high classification accuracy while ensuring wide-range classification. Our method solve the matching problem between large model platforms and the deep learning models. Furthermore, we improve the accuracy of dataset prediction and label classification, which is higher than that of the existing fusion methods. Our method can be deployed on small devices, which is important for many applications.

In future work, we will conduct more experiments to study how the diversity of samples affects the performance of trained models, aiming to further increase classification accuracy. Furthermore, as the same wrong results lower the performance of the fusion methods, the similarity of trained models will also be a focus for future research.
